# Urban gardens promote bee foraging over natural habitats and plantations

**DOI:** 10.1002/ece3.1941

**Published:** 2016-01-28

**Authors:** Benjamin F. Kaluza, Helen Wallace, Tim A. Heard, Alexandra‐Maria Klein, Sara D. Leonhardt

**Affiliations:** ^1^ Institute of EcologyLeuphana University21335LüneburgGermany; ^2^ Faculty of Science, Health, Education and EngineeringUniversity of the Sunshine CoastMaroochydore4558Australia; ^3^ Department of Animal Ecology and Tropical BiologyUniversity of Würzburg97074WürzburgGermany; ^4^ CSIRO Ecosystem SciencesBrisbane4001QldAustralia; ^5^ Chair of Nature Nature Conservation and Landscape EcologyUniversity of Freiburg79085FreiburgGermany

**Keywords:** Anthropogenic activities, climate factors, meliponines, resource availability, urbanization

## Abstract

Increasing human land use for agriculture and housing leads to the loss of natural habitat and to widespread declines in wild bees. Bee foraging dynamics and fitness depend on the availability of resources in the surrounding landscape, but how precisely landscape related resource differences affect bee foraging patterns remains unclear. To investigate how landscape and its interaction with season and weather drive foraging and resource intake in social bees, we experimentally compared foraging activity, the allocation of foragers to different resources (pollen, nectar, and resin) and overall resource intake in the Australian stingless bee *Tetragonula carbonaria* (Apidae, Meliponini). Bee colonies were monitored in different seasons over two years. We compared foraging patterns and resource intake between the bees' natural habitat (forests) and two landscapes differently altered by humans (suburban gardens and agricultural macadamia plantations). We found foraging activity as well as pollen and nectar forager numbers to be highest in suburban gardens, intermediate in forests and low in plantations. Foraging patterns further differed between seasons, but seasonal variations strongly differed between landscapes. Sugar and pollen intake was low in plantations, but contrary with our predictions, it was even higher in gardens than in forests. In contrast, resin intake was similar across landscapes. Consequently, differences in resource availability between natural and altered landscapes strongly affect foraging patterns and thus resource intake in social bees. While agricultural monocultures largely reduce foraging success, suburban gardens can increase resource intake well above rates found in natural habitats of bees, indicating that human activities can both decrease and increase the availability of resources in a landscape and thus reduce or enhance bee fitness.

## Introduction

Animal pollination is a key ecosystem function, and modern agriculture benefits from pollinators, particularly bees, for the production of many crops (Klein et al. 2007; Garibaldi et al. 2013). Reports of declines in managed and wild bees thus raise concerns about a global pollination crisis (Allen‐Wardell et al. 1998; Winfree 2010). Bee pollinators are under pressure from human activities (Winfree 2010), and bee decline is often linked to habitat change and loss (Winfree et al. 2009; Potts et al. 2010; Vanbergen & the Insect Pollinators Initiative 2013). Many natural habitats have been destroyed or fragmented by urbanization and agricultural intensification with parallel declines observed in the diversity and abundance of insect pollinators (Aizen and Feinsinger 1994; Steffan‐Dewenter et al. 2002; Ricketts 2004; Vanbergen & the Insect Pollinators Initiative 2013). Anthropogenic changes to habitat may confound underlying and interacting effects that regulate bee populations, such as food resource availability (Roulston and Goodell 2011). How landscape related differences in resource availability affect foraging patterns and resource intake of bees has however received little attention.

Bees typically find a constant supply of floral resources in (semi‐)natural habitats, which provide a high diversity of plants (Cairns et al. 2005; Rundlöf et al. 2008; Roulston and Goodell 2011; Kennedy et al. 2013). In contrast, in intensively managed agricultural monocultures, food resources are only abundant during the short flowering seasons of crops (Decourtye et al. 2010). Subsequent shortages in food resources throughout the rest of the year have been linked to honey bee colony collapses in degraded habitats (Naug 2009). Urban areas may, on the other hand, also provide steady food resources throughout the year due to the presence of many native and exotic plant species in gardens (Loram et al. 2008; Roulston and Goodell 2011). However, foraging patterns and resource intake of bees in urban landscapes such as gardens have, to our knowledge, not yet been studied.

Highly social bees form long‐lived colonies and thus need floral resources throughout the entire season. Foraging activity on the colony level is regulated by (1) the amount of resources stored within the nest and (2) the availability of resources in the environment (Biesmeijer et al. 1999; Hofstede and Sommeijer 2006; Altaye et al. 2010). Foraging activity and patterns of colonies with similar food storages, but located in different environments, should therefore be mainly determined by the availability of resources in the respective landscapes.

Bees collect a variety of plant resources, primarily floral nectar and pollen (Michener 2007; Brodschneider and Crailsheim 2010). Nectar is the main energy source for bees and pollen provides the proteins, lipids, vitamins, and minerals crucial for brood rearing, but is also consumed by adult bees (Nicolson 2011). Highly social bees, such as tropical stingless bees (Apidae: Meliponini) and honey bees, collect resin as additional plant resource, predominantly from wounded trees (Roubik 1989). Resin is used for nest construction and defence against predators or parasites (Leonhardt and Blüthgen 2009; Greco et al. 2010) and is essential for colony survival. Bees therefore need to divide their foraging efforts between these different plant resources.

Foraging behavior and daily flight activity of bees is further influenced by abiotic factors, such as temperature, humidity, solar radiation, and wind (Heard and Hendrikz 1993; Hilário et al. 2012; Oliveira et al. 2012; Polatto et al. 2014). Variations in weather and resource availability can therefore differentially affect foraging activity depending on the season (Ferreira et al. 2010; Figueiredo‐Mecca et al. 2013). Whether weather factors or resource availability in a landscape predominately shape the foraging behavior of bees is however still unclear.

We compared foraging patterns, i.e. forager allocation and foraging activity, and resource intake of a common Australian stingless bee species, *Tetragonula carbonaria* Smith, between plantations, forests, and suburban gardens. Our aim was to better understand how differently altered human landscapes, i.e. agricultural areas and gardens, affect resource foraging in highly social bees compared with patterns observed in their natural habitat.

We specifically addressed the following questions:

1. How do different landscapes, altered and natural, influence foraging patterns, i.e. foraging activity, forager numbers and proportions of bees collecting different floral resources, in a generalist social bee?

We predict foraging patterns to be influenced by long periods of food shortages in agricultural landscapes (Decourtye et al. 2010), resulting in low activity and forager numbers throughout most of the year except for the short macadamia flowering period. We further predict foraging activity and numbers to be intermediate in gardens due to a constant but patchy distribution of resources, and to be highest in natural landscapes due to year‐long availability of abundant resources. Allocation of foragers to different resources (i.e. forager proportions) is expected to be similar across landscapes and seasons for pollen and nectar, while the number of unsuccesful foragers should be high in plantations and low in forests. Due to the higher abundance of trees in forests, we expect our colonies to allocate more foragers to resin collection in forests than in gardens and plantations.

How does sugar and pollen intake by social bees differ between different landscapes?

Overall resource intake is predicted to increase in landscapes comparatively richer in plant resources, such as forests and gardens, and be highest in their natural habitat (forests).

How do abiotic factors (e.g. temperature, humidity, wind) interact with landscape in determining foraging activity and patterns?

We predict that abiotic factors contribute to foraging patterns, but that foraging patterns are mainly determined by landscape.

## Methods

### Study species and landscapes

The study was conducted in Queensland, Australia. We chose the Australian stingless bee *Tetragonula carbonaria* as a model species to address our research questions (Dollin et al. 1997; genus change: Rasmussen and Cameron 2007). *Tetragonula carbonaria* occurs as a wild bee and native pollinator in the study region, and can also be kept and propagated in boxes and thus be managed for crop pollination (Heard and Hendrikz 1993; Heard 2016). This allows colonies to be placed in specific landscapes and to experimentally test for the effect of habitat and landscape on a perennial bee species.

Observations were conducted within the native range of the species in Queensland. The East coast of Queensland is characterized by a subtropical climate with wet summer and dry winter seasons. To test how colonies of *T. carbonaria* were influenced by resource diversity and availability in different landscapes, we selected three landscape types characteristic of the region to experimentally place hives of *T. carbonaria*: forests, plantations, and gardens.

Forests ranged from relatively open *Banksia* heathland to more dense forests with closed canopy, but were all dominated by an overstory of *Eucalyptus* and *Corymbia* species and thus reflected the variety of habitats commonly used by *T. carbonaria* (Dollin et al. 1997). Australian forests have been historically shaped by dynamic processes like anthropogenic fire regimes and are continuously exposed to moderate disturbance (Bird et al. 2008). Thus, uncleared forests, as selected in this study, can be considered a natural environment. Before we started our study, we confirmed that wild colonies of *T. carbonaria* were present at all forest study sites to ensure that the forest sites represent valid natural control sites.

Our plantation sites were represented by commercial macadamia plantations (*Macadamia integrifolia* Maiden and Betche X* M. tetraphylla* Johnson). Macadamia are indigenous rainforest trees grown for their edible nuts, and are known to be pollinated by *T. carbonaria* (Vithanage and Ironside 1986; Heard 1994; Heard and Exley 1994). All plantations were monocultures with at most ten different genotypes as commercial macadamia varieties are genetic clones.

We additionally placed bee hives in another human altered landscape, suburban gardens, a habitat which has been successfully used to breed stingless bees by private bee enthusiasts in Australia (Klumpp 2007). Suburban gardens in the study region typically include houses, surrounded by gardens of 300–1000 m^2^ with native and exotic plants. Exotic plants, i.e. introduced alien plant species as well as ornamental cultivars, commonly made up more than 50% of all garden plant species in our study (data not shown). Gardens were mostly situated in suburbs with remnants of uncleared bush vegetation or small parks with mature *Eucalyptus* or other native trees.

### Experimental setup

**Figure 1:**
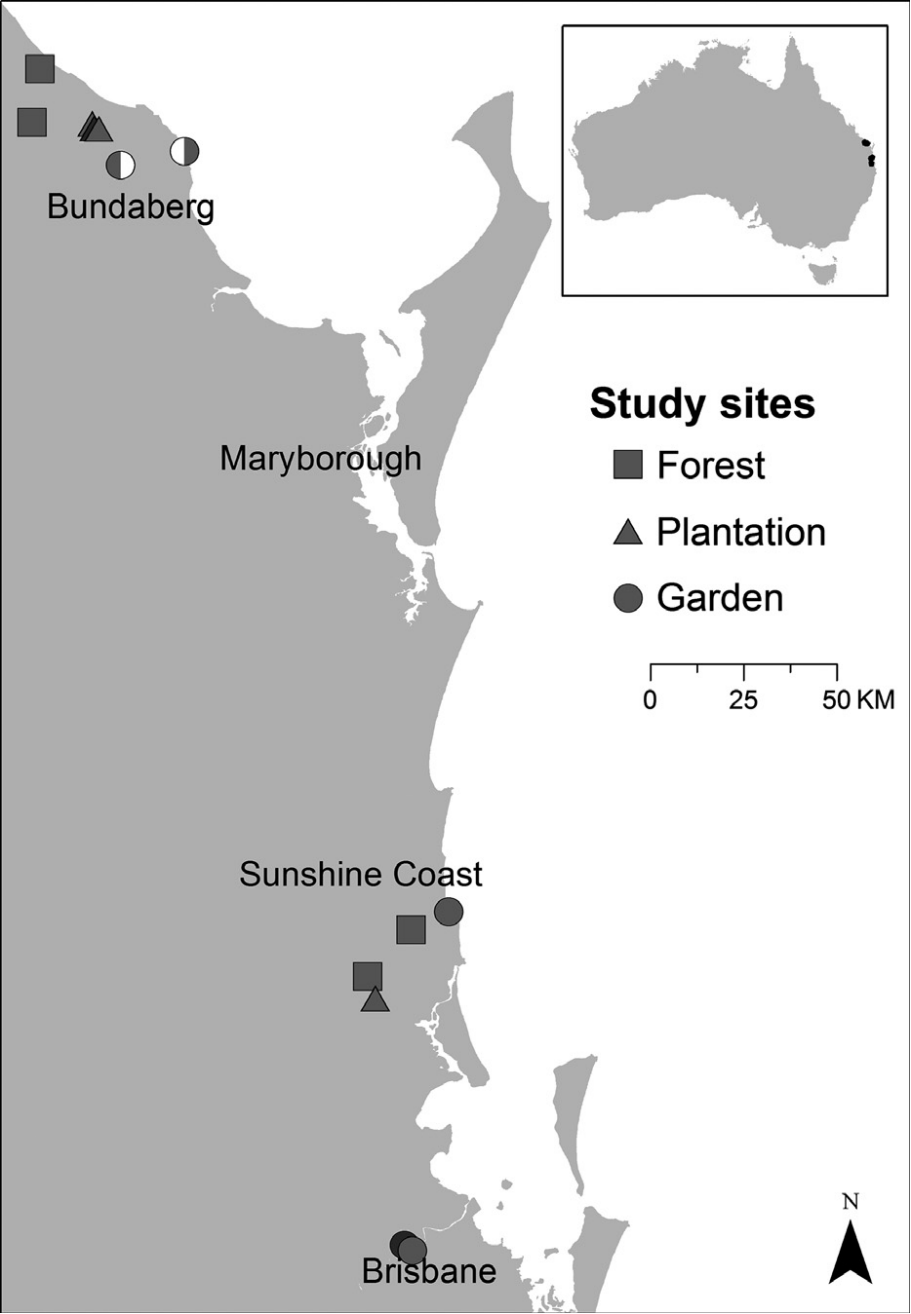
Location of study sites in South East Queensland, Australia. Study sites of each landscape category (plantation, forest, garden) were established at three different regions, ranging from Bundaberg (north) to the Sunshine Coast area and Brisbane (south). Half‐filled circles represent the two locations (each with two hives) of one garden site in Bundaberg.

A total of 12 study sites were established in 2011 in two regions in South East Queensland, ranging from the Bundaberg region in the north to the Sunshine Coast area and Brisbane region in the south (Fig. 1, Table S1; 24°38′‐27°30′S, 152°6′‐153°7′E). For each landscape type (plantation, forest and garden) we chose four study sites as replicates, with replicates of each landscape in the northern and southern region to avoid spatial autocorrelation. At each study site, we placed four wooden bee hives containing *T. carbonaria*. Consequently, a total of 48 *T. carbonaria* bee hives were set up at all study sites in 2011.

In gardens, space was limited and hives needed to be distributed among two suitable private garden locations in close proximity (mean ± SD distance: 706 ± 129 m, except for one garden site with 16 km between garden locations, Fig. 1). Two hives were placed on each location and both garden locations together were considered one garden site. We allowed for a 500 m flight radius of the bees around the hives which is considered the typical foraging range of bees of this size (Greenleaf et al. 2007, equivalent to 0.78 km² flight range). We further made sure that flight ranges of different study sites did not overlap (sites separated by > 1.1 km in plantations, > 14.3 km in forests and > 1.4 km in gardens). To ensure that more than 75 % of the flight range was covered by the target landscape (plantation, forest or garden) we evaluated the vegetation cover by aerial photographs from Google Earth. We outlined all vegetation patches to calculate their area with the software KML Toolbox. All vegetation patches were additionally validated by ground surveys.

Bee hives were mounted on metal posts 1 m above ground (in forests and plantations), orientated with the entrance facing NE, or placed on bricks low above ground where sealed surfaces did not allow the use of posts (in gardens). All hives were placed with a minimum distance of 5 m in between and in shaded or semishaded locations and protected by a metal roof where no other cover was available. Our study hives were all provided by T. Heard. They had not been disturbed for at least 3 months prior to the setup and had comparable starting weights of 7.2 ± 0.7 kg (= combined weight of colonies and hive boxes). In plantations, hives were closed and covered for at least 24 h when insecticides were applied to macadamia trees to prevent contamination of hives.

Nest densities in the experiment were similar to those found in Australian forests and suburban areas, i.e. typically 1 up to 3 colonies/ha (Heard 2016), and comparable to nest densities found for other stingless bee species in Australia and Borneo (Eltz et al. 2002; Halcroft 2012). The foraging behavior of our hives should thus not be influenced by increased competition for food resources.

### Observations of foraging patterns

To study how the three landscape types affected foraging patterns, activity, and resource intake of hives, we observed foraging bees from September 2011 to September 2013. To account for seasonal differences in foraging behavior, foraging observations were carried out in three seasons per year over 2 years: in the dry season (September‐December), wet season (January‐April) and cold season (May‐August). In each season, observations of each hive were repeated on three different days to account for changing weather conditions. Each hive was revisited within 12 ± 9 days and all hives of targeted study sites were visited at least once within 31 ± 9 days. For each landscape type, two sites were selected for the foraging observations (one in the northern and one in the central region of the study area). At each site, 3–4 hives were observed per season (summing up to a total of 18–24 hives at overall six study sites). Overall, we assembled a data set with 9950 recorded foraging trips for 512 hive observations.

Observations were conducted between 7:30 and 15:30 on rain‐free days (see Data S1). The following weather conditions were recorded for each observation period: ambient temperature, humidity (PCE‐555 Digital Psychrometer; PCE Instruments, Meschede, Germany), and cloud cover (estimated in 12.5% steps of covered sky). In the second year, we also recorded wind conditions (average and maximum in m/sec and gustiness: number of wind peaks/3 min; PCE‐MAM 1 anemometer, PCE Instruments, Meschede, Germany).

The overall foraging activity of each hive was recorded first by counting the number of returning foragers for 3 min. Then 20 returning foragers were captured and their pollen, nectar or resin load visually inspected and counted to assess the total number and proportion of respective foragers as well as unsuccessful foragers (Leonhardt et al. 2014). To calculate forager numbers per minute for each resource, respective proportions were multiplied by activity. All foragers captured were held until the end of the observation period to avoid recapturing the same individual.

### Resource intake

Nectar foragers were identified by their swollen abdomen. To collect the nectar, their abdomen was carefully squeezed to provoke regurgitation of the crop content. Nectar volume was quantified in 5 *μ*l microcapillary tubes (Camag, Muttenz, Switzerland) and nectar concentration was measured to the nearest 0.5 g/g sucrose equivalent by hand‐held refractometers (Eclipse Refractometer; Bellingham + Stanley Ltd., Lawrenceville, GA). The sugar concentration in nectar (c in %) was converted into *x* (in *μ*g/*μ*L) following (Kearns Blüthgen, N. and Inouye 1993) with the values adjusted by Blüthgen (pers. commun.) according to the equation:

$$x=-0.0928+10.0131*c+0.0363*c^{2}+0.0002*c^{3}.$$

With *x* and the measured nectar volume (*V*) we calculated the sugar load of each individual nectar forager (in mg). To calculate the average sugar intake (in mg/min) for each hive observation the following equation was applied:

$$\frac{\sum_{1}^{n}\left(x*V\right)*A*P_{N}}{n}$$

where *n* is the overall number of nectar foragers for a given hive and observation, *A* the hive activity, and *P*_*N*_ the corresponding proportion of nectar foragers.

Pollen loads of foragers were removed from each hind leg with forceps and collected in previously weighed Eppendorf tubes. The two pollen loads of each leg of a forager were collected in two separate Eppendorf tubes. Eppendorf tubes were reweighed after inserting pollen to calculate the average net pollen weight carried by all foragers. The total pollen intake per minute of each hive (in mg/min) was then calculated as follows:

$$\frac{\left(E_{1}+E_{2}\right)*A*P_{P}}{n}$$

with *E*_*1*_ and *E*_*2*_ as the net pollen weights in each Eppendorf tube, *n* the number of captured pollen foragers, *A* the activity of the hive per minute and *P*_*P*_ the proportion of pollen foragers for this observation period.

### Statistical analysis

We used generalized linear mixed effect models (GLMM) to analyse the effects of landscape type, season and weather variables (explanatory variables) on foraging activity, pollen, nectar, resin, and unsuccessful forager proportions and numbers, as well as nectar concentration, sugar, and pollen intake (response variables; R‐Development‐Core‐Team 2009; library lme4: Bates et al. 2011). As we collected data from several hives located at several study sites for each landscape, hive nested within site was entered as a random effect in all models. Landscape (plantation, forest, garden) and season (dry, wet, and cold season) were entered as fixed categorical variables.

To test effects of landscape and season on the proportion of pollen, nectar and resin foragers, forager numbers were entered as a binomial vector, i.e. a two‐column matrix with the columns giving the numbers of successes (e.g. number of pollen foragers) and failures (e.g. number of non‐pollen foragers) using GLMMs with a binomial error distribution. Pollen, nectar, resin, or unsuccessful foragers per minute as well as total sugar intake did not show a Gaussian distribution, even when response variables were transformed, and we therefore applied GLMMs with a Poisson distribution. Total pollen intake per minute showed over‐dispersion and was thus square‐root transformed and analysed with GLMMs with a Poisson distribution. Nectar concentration was arcsine square‐root transformed.

For each response variable, different models were composed, starting with the most complex model (including all explanatory variables and interactions between them). Next, we stepwise dropped interactions between explanatory variables and then variables (wind, temperature, season, and landscape type). The quality of all models was compared using Akaike's Information Criterion (AIC) and the model with the lowest AIC value was considered the model with the highest explanatory value. To test whether individual explanatory variables in the model with the lowest AIC value actually explained a significant proportion of the overall variance, we compared the model with a given variable to the same model without this variable using the ANOVA command in the lme4 package which compares two nested models based on likelihood‐ratio tests and chi‐square statistics. For models with landscape as significant explanatory variable, differences between landscape types were further evaluated using Tukey's post hoc test (package multcomp: Hothorn et al. 2008).

To test how weather affected foraging patterns and interacts with landscape, we performed a second set of models with the weather variables (i.e. temperature, humidity, wind, cloud cover) included. To account for collinearity of weather variables, we created a Spearman rank correlation matrix, which revealed two clusters of variables (a: temperature, humidity, and cloud cover; b: wind gusts, average and maximum wind speed, see Table S2). From those we selected temperature and average wind speed to test their influence on our response variables in the models. Note that comprehensive weather variables were only available for a smaller subset of the data and therefore analysed for this data set only to avoid the loss of degrees of freedom (compare Table 1 and Table S3), which in combination with the reduction of the data sets limits the explanatory power of the analysis.

**Table 1:** Results of generalized linear mixed effect models (GLMMs) for each response variable. Given are *χ* ^2^‐values and degrees of freedom (df) obtained for comparing the best model with the respective explanatory variable to a model with this variable dropped (landscape, season) and the interaction of both factors. Significance levels as follows: *P <* *0.05, **P <* *0.01, ***P <* *0.001, ns not significant

Response variable	Landscape			Season			Interaction		
	*χ* ^*2*^	df	*P*	*χ* ^*2*^	df	*P*	*χ* ^*2*^	df	*P*
Foraging activity	8.88	2	*			ns			ns
Pollen foragers/min	171.54	6	***	259.67	6	***	160.94	4	***
Nectar foragers/min	150.27	6	***	164.81	6	***	139.74	4	***
Resin foragers/min	86.89	6	***	86.01	6	***	83.08	4	***
Unsuccessful foragers/min	122.43	6	***	192.89	6	***	119.95	4	***
Proportion pollen foragers	54.83	6	***	114.57	6	***	50.44	4	***
Proportion nectar foragers	101.77	6	***	196.27	6	***	100.19	4	***
Proportion resin foragers	37.04	6	***	53.10	6	***	34.65	4	***
Proportion unsuccessful foragers	56.81	6	***	101.25	6	***	48.45	4	***
Sucrose concentration in nectar	23.01	6	***	205.42	6	***	18.95	4	***
Total sugar intake/min	187,699	6	***	316,369	6	***	187,685	4	***
Pollen load size			ns	43.17	2	***			ns
Total pollen intake/min	9.45	2	**	10.05	2	**			ns

## Results

### Foraging patterns

Differences in bee foraging activity were best explained by landscape, without any other explanatory factors contributing significantly (Table 1). Across seasons, foraging activity was highest in gardens, lower in forests and lowest in macadamia plantations (mean activity ± SD in plantations: 17 ± 17; forests: 27 ± 19; gardens: 38 ± 26 foragers/min), with a significant difference between gardens and plantations (Tukey test, *P* = 0.004).

**Figure 2:**
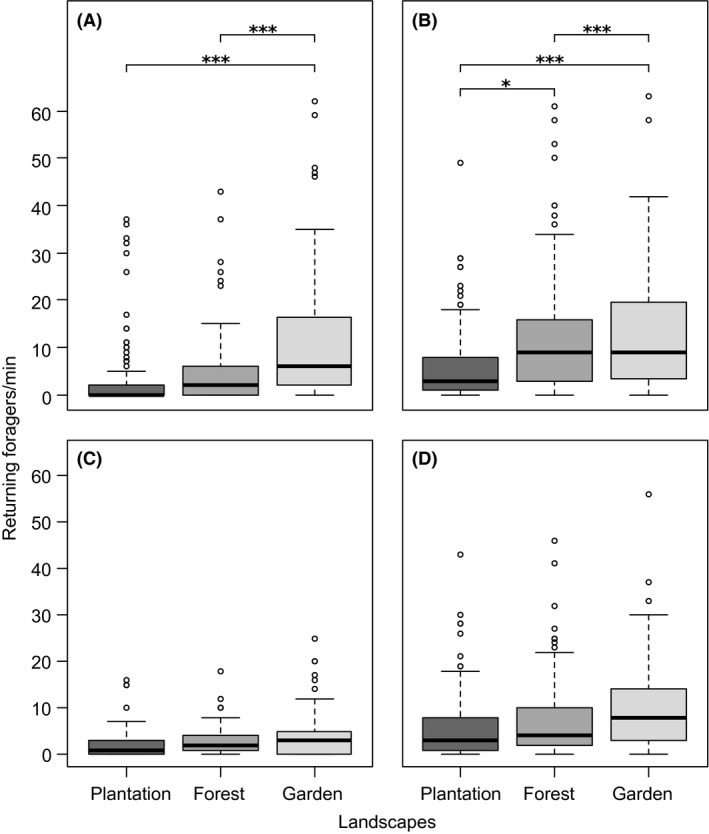
Number of foragers per minute returning with (A) pollen, (B) nectar, (C) resin, and (D) unsuccessful foragers in plantations (dark gray bars), forests (gray) and gardens (light gray). Boxplots display the median (thick bar), lower (0.25) and upper (0.75) quartile (gray box), minimum and maximum values (whiskers) and outliers of each dataset. Asterisks indicate significant differences between landscapes according to Tukey's posthoc test, significance levels as follows: *P <* *0.05, **P <* *0.01, ***P <* *0.001.

Differences in the number of foragers for all resources (pollen, nectar, and resin) were best explained by the interaction between landscape and season (Table 1, Fig. S1). That is to say, resource foraging showed different seasonal patterns in different landscapes, e.g. pollen, nectar and resin forager numbers were significantly highest in gardens in the wet but not in the cold or dry season (Fig. S1). Across seasons, significantly more pollen foragers returned to the hive per minute in gardens than in both forests and plantations (Fig. 2A), while nectar foragers were high in both forests and gardens (Fig. 2B). However, nectar foragers differed between forests and gardens in their seasonal patterns, as nectar foragers tended to be highest in forests in the cold season, but tended to be highest in gardens in the dry season (Fig. S1). Numbers of resin and unsuccessful foragers did not differ between landscapes (Fig. 2C,D).

**Figure 3:**
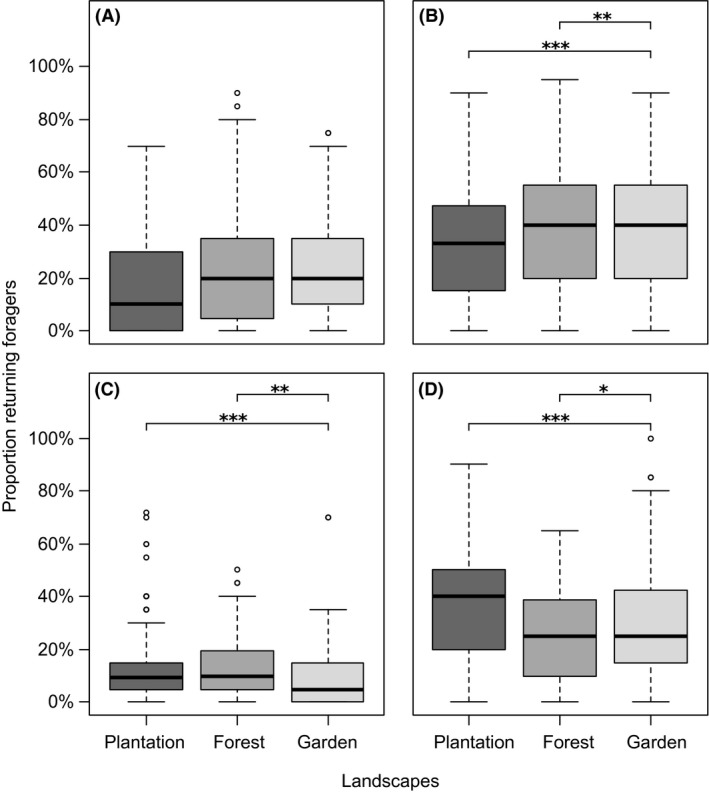
Proportional resource intake in plantations (dark gray bars), forests (gray) and gardens (light gray). Shown are per cent of foragers returning with (A) pollen, (B) nectar, (C) resin, and (D) unsuccessful foragers. Boxplots display the median (thick bar), lower (0.25) and upper (0.75) quartile (gray box), minimum and maximum values (whiskers) and outliers of each dataset. Asterisks indicate significant differences between landscapes according to Tukey's posthoc test, significance levels as follows: *P <* *0.05, **P <* *0.01, ***P <* *0.001.

The interaction between landscape and season also best explained differences in forager proportions for all foraging resources (Table 1, Fig. S2). Across seasons, hives located at different landscapes allocated similar proportions of bees to pollen foraging (Fig. 3A), but pollen foraging patterns strongly differed between landscapes for different seasons. For instance, in the wet season, pollen forager proportions were significantly highest in gardens and lowest in plantations, whereas the pattern tended to be reversed in the dry season (Fig. S2). The proportion of nectar foragers was generally high in gardens and forests (plantations: 33 ± 23%; forests: 40 ± 23%; gardens: 37 ± 23%; Fig. 3B), but showed the same inversed seasonal trends in forests and gardens as nectar forager numbers (Fig. S2). Proportions of resin foragers were overall low in gardens compared to forests and plantations (Fig. 3C), but did not differ between landscapes in the cold season (Fig. S2). Plantations had the significantly highest proportion of unsuccessful foragers in all seasons (Fig. 3D), but while the proportion of unsuccessful foragers was by trend lowest in gardens in the dry season, it tended to be lowest in forests in the cold season (Fig. S2).

### Resource intake

Differences in sugar concentration were best explained by the interaction of landscape and season (Table 1). For more than half of our observations, sucrose concentration in nectar collected by foragers ranged between 60 and 75% (total *N* = 2647) and did not significantly differ between landscapes (mean sucrose concentration in plantations: 57.85 ± 13.61%; forests: 52.56 ± 14.22%; gardens: 55.83 ± 14.84%). However, nectar sugar concentration varied over the year (Fig. S3) and was higher in the dry than in the wet and cold season (Tukey test, *P* < 0.001; dry season: 65.32 ± 13.63%; wet season: 52.60 ± 18.81%; cold season: 55.13 ± 14.69%).

**Figure 4:**
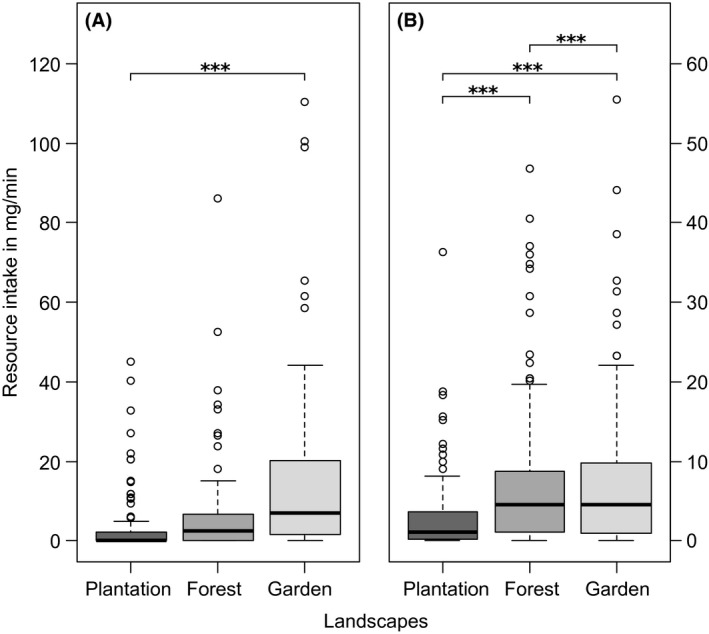
Pollen (A) and sucrose (B) intake in plantations (dark gray bars), forests (gray) and gardens (light gray). Boxplots display the median (thick bar), lower (0.25) and upper (0.75) quartile (gray box), minimum and maximum values (whiskers) and outliers of each dataset. Asterisks indicate significant differences between landscapes according to Tukey's posthoc test, significance levels as follows: * *P *<* *0.05, ** *P *<* *0.01, *** *P *<* *0.001.

Whereas pollen load size of individual workers did not differ between landscapes (plantations: 1.13 ± 0.56 mg; forests: 1.15 ± 0.33 mg; gardens: 1.26 ± 0.47 mg), it did differ between seasons (Fig. S3; GLMM: *χ* ^2^ = 43.17, *P* < 0.001) and was overall highest in the wet season (1.32 ± 0.46 mg) and lowest in the cold season (0.91 ± 0.29 mg) and intermediate in the dry season (1.01 ± 0.44 mg). Landscape and season also best described differences in the total pollen intake per minute (Table 1, Fig. S4). Total pollen intake per minute of the whole colony was overall lowest in plantations and significantly higher in forests and gardens (Fig. 4A).

Differences in the total sugar intake per minute were also best described by the interaction between landscape and season (Table 1, Fig. S4). Sucrose intake per minute was generally high in gardens and forests and significantly lower in plantations (Fig. 4B), like the seasonal patterns of nectar foragers (Fig. 2B, Fig. S1).

### Influence of weather

Weather variables, i.e. temperature and average wind speed, significantly affected our foraging response variables (Table S3). However, variation in foraging activity and forager numbers was largely explained by interactions between landscape, season, temperature, and average wind speed (Table S3). The same was true for proportions of pollen, nectar, and unsuccessful foragers as well as total pollen and sugar intake (Table S3). The proportion of resin foragers was not influenced by temperature, and resin foragers per minute were not influenced by average wind speed (Table S3). Consequently, landscape had a strong influence in all models even when weather variables were included, and the explanatory values of all models significantly decreased when landscape was dropped (GLMM: *P* < 0.001 in all cases, Table S3).

## Discussion

Wild bee populations are declining in human altered landscapes likely due to reduced availability of food resources (Decourtye et al. 2010; Winfree 2010; Roulston and Goodell 2011). Because plant resource availability and diversity in landscapes drive foraging dynamics in bees (Decourtye et al. 2010; Jha and Kremen 2013), we investigated how foraging patterns and resource intake in a highly social bee species are affected by landscape related differences in resource availability. Our results clearly show that foraging patterns strongly differed between different human altered landscapes and the bees' natural habitat depending on season. Contrary with our expectations, pollen and nectar foraging, nectar forager numbers and sugar and pollen intake were highest in gardens, not in natural forests.

### Foraging patterns

Foraging activities were highest in gardens across all seasons in both years, indicating that gardens provide abundant floral resources to forage on compared with other landscapes. All key resources needed for provison and rearing brood were abundant and fully utilized by bee hives in gardens. The steady food availability was most likely due to a mix of native and exotic plants in gardens which produce a continuous supply of floral resources (Head et al. 2004), known to benefit generalist bee species (Winfree 2010; Levy 2011). This result agrees with previous findings showing that urban or suburban gardens represent beneficial landscape elements by providing plentiful food resources and foraging opportunities for bees which increases bee abundance and density in social and solitary bees (Gotlieb et al. 2011; Samnegård et al. 2011; Hinners et al. 2012). Moreover, access to anthropogenically disturbed patches with additionally planted (flowering) plant species in a homogenous natural landscape can improve habitat quality, as connected patches of high plant diversity in a mosaic landscape provide additional foraging opportunities (Williams and Kremen 2007; Winfree et al. 2007). Human altered, highly heterogenous habitats, such as gardens, can consequently be of high foraging value. While Hernandez et al. (2009) suggest that this positive effect of urbanization may be limited to eusocial or generalist bees, Baldock et al. (2015) found bee richness across taxa to be higher in urban areas than on farms and to be marginally higher in urban areas than in nature reserves.

Social bee colonies further respond to the spatio‐temporal changes of resource availability in a landscape by adjusting the number of foragers for any target resource according to their colony needs. We found high proportions of nectar foragers and lower proportions of resin and unsuccessful foragers in gardens than in other landscapes, whereas the proportion of pollen foragers did not differ between landscapes. Pollen is a limited plant resource and is, unlike nectar, not constantly replenished by the plant and can thus be depleted over the course of a day (Roubik 1989). Bees should thus primarily collect pollen when available. Periods of high pollen availability occurred at all of our study sites. Consequently and as predicted, we found a similar proportion of pollen foragers when comparing landscapes across seasons.

The generally higher proportion of successful foragers in gardens is most likely due to the very small‐scaled and patchy resource landscape with steady flowering across all seasons, including a variety of bird pollinated native plants with a continuous supply of nectar (Ford et al. 1979). Contrary with our predictions, resin foraging was not higher in forests than plantations, even though resin availability was predicted to largely increase with tree availability (Leonhardt and Blüthgen 2009). In gardens with limited numbers of resiniferous trees, hives allocated a smaller proportion of foragers, but similar overall forager numbers to collect resin. Stingless bee workers are known to rarely switch from or to resin foraging behavior during the day, which keeps resin forager numbers fairly steady (Inoue et al. 1985; Wallace and Lee 2010). An overall higher foraging activity in gardens therefore allows hives to collect more pollen and nectar, while gathering similar total amounts of resin, compared to hives with lower foraging activities in forests or plantations. Contrary with our expectations, *T. carbonaria* thus seemed to have a specific intake target for resin as we observed similar numbers of returning resin foragers in all landscapes, which contradicts our prediction and suggests that resin is sufficiently available in all landscapes.

In contrast with gardens with their continuous pollen supply, pollen collection as well as overall foraging activity in forests seemed to be largely driven by the main mass flowering of eucalypts in the dry and cold season (Beardsell et al. 1993). The effect of mass flowering on a colony's pollen intake has also been shown for stingless bee colonies in Borneo which strongly responded to the mass flowering of dipterocarp trees (Eltz et al. 2001). Mass flowering crops also increase foraging and reproductive success in honey bees and solitary bees (Jauker et al. 2012; Odoux et al. 2012).

In accordance with our expectations, the number of unsuccessful foragers was high in plantations and foraging activity generally weak and only peaked during the 5–8 week period of macadamia mass flowering in the dry season (Heard 1993; Wallace et al. 1996). But even then, it rarely reached as high activity levels as observed in gardens. Plantation hives may have struggled to build up sufficient numbers of foragers to make use of the macadamia mass flowering after a long dormant state in the cold season. Foraging nevertheless continued all year long in plantations, but limited availability of flowering plants besides macadamia strongly constrained foraging activity of hives. This finding agrees with previous studies showing that seasonal resource limitation impacts on bee foraging in landscapes with mass flowering crops dominating (Decourtye et al. 2010; Williams et al. 2012).

### Resource intake

Sucrose concentrations between 60–75%, as often observed in our study, are unusually high compared to other ecosystems with maximum concentrations of 60% or often <35% sugar content of nectar collected by bees (Roubik 1989). Australia and specifically its arid areas have been proposed to offer plentiful carbohydrate resources, which in turn favor opportunistic social insects (Morton et al. 2011). We found highest nectar concentrations in the dry season across landscapes which further points to the importance of short flowering events of specific nectar plants, e.g. macadamia or eucalypts, as a driver of nectar foraging dynamics. Although the nectar collected likely originated from different foraging plant sources in the different landscapes, nectar of high quality seemed to be available in all landscape types and does not explain resource related shortcomings.

Sugar intake rates were nevertheless two to three times higher in gardens and forests than in plantations, with greatest differences between landscapes in the dry season. As nectar concentration varied little between landscapes and season, sugar intake rates were predominantly determined by the overall proportion of nectar foragers and hive foraging activity.

Pollen intake rates of hives in forests were twice as high as in plantations and five times higher in gardens than in plantations. Yet the size of pollen loads of single workers, which corresponds to the efficiency of single foraging trips, did not vary between landscapes across seasons. Pollen foragers were thus likely able to maximize their load in all landscapes. Consequently, the higher pollen foraging success in forests and gardens was again due to higher foraging activity. This finding highlights the role of hive foraging activity as a response to landscape resource availability in determining the overall foraging success of social bees.

Unlike social bees, generalist solitary bees cannot equivalently increase their resource intake in response to increasing resource availability, because they cannot recruit additional bees to foraging when resources are plentiful. Thus, even if they could use all plant sources available to social bees, their abundance and fitness would most likely be more strongly affected by other parameters, such as foraging distances (Zurbuchen et al. 2010) or climate (Vicens and Bosch 2000) provided they have access to sufficient nesting opportunities (Zanette et al. 2005; Cane et al. 2006; Hernandez et al. 2009).

Abiotic factors, like temperature, humidity, wind speed, and luminosity, are known to further strongly influence bee foraging behavior, especially in tropical stingless bees (Ferreira et al. 2010; Figueiredo‐Mecca et al. 2013) and other bees (Brittain et al. 2013; Kühsel and Blüthgen 2015). These weather factors also contributed to the activity patterns observed in our study, but their influence was minor compared to landscape related patterns of resource foraging.

To summarize, we found that landscape strongly affected foraging patterns and resource intake in a social bee. Moreover, bees responded differently to different anthropogenic habitat alterations compared to natural forest habitats, with foraging activity and thus resource intake being strongly impaired in agricultural monocultures, but largely improved in flower‐rich gardens. While previous studies focused on the negative effects of plant resource impoverishment in agricultural landscapes on bees (Decourtye et al. 2010; Lentini et al. 2012; Williams et al. 2012), few studies have hitherto investigated how gardens affect bee foraging and resource intake (Hennig and Ghazoul 2012; Wojcik and McBride 2012). Cities worldwide differ in the extent of remaining green areas, flower resources and nesting space and may thus differentially affect bees (Hernandez et al. 2009; Matteson et al. 2013; Lowenstein et al. 2014), but our study shows that gardens can increase resource intake and thus foraging success in social bees even beyond natural habitats.

## Conflict of Interest

None declared.

## Supporting information


**Table S1.** Location of study sites and geographic information.
**Data S1.** Influence of daytime.
**Table S2.** Spearman correlation matrix with correlation coefficients (r_S_) for forager numbers and weather variables.
**Table S3.** Results of generalized linear mixed effect models (GLMMs) for each response variable, for the second year with all weather factors included as additional explanatory variables.
**Figure S1.** Number of foragers returning per minute with pollen, nectar, resin or unsuccessful foragers in plantations, forests and gardens in the wet, cold and dry season.
**Figure S2.** Proportional resource intake in plantations, forests and gardens in the wet, cold and dry season.
**Figure S3.** Hive foraging activity, pollen loads and sucrose concentration of nectar in plantations, forests and gardens in the wet, cold and dry season.
**Figure S4.** Pollen and sucrose intake in plantations, forests and gardens in the wet, cold and dry season.
**Data S2.** Results of generalized linear mixed effect models (exported from R Statistics).
