# Supplementary material for: Urban gardens promote bee foraging over natural habitats and plantations
Source: Ecol Evol. 2016 Jan 28;6(5):1304–16. doi: 10.1002/ece3.1941 (PMC4730924; doi:10.1002/ece3.1941)
Supplement: Supplementary file 1 — Table S1. Location of study sites and geographic information. Data S1. Influence of daytime. Table S2. Spearman correlation matrix with correlation coefficients (rS) for forager numbers and weather variables. Table S3. Results of generalized linear mixed effect models (GLMMs) for each response variable, for the second year with all weather factors included as additional explanatory variables. Figure S1. Number of foragers returning per minute with pollen, nectar, resin or unsuccessful foragers in plantations, forests and gardens in the wet, cold and dry season. Figure S2. Proportional resource intake in plantations, forests and gardens in the wet, cold and dry season. Figure S3. Hive foraging activity, pollen loads and sucrose concentration of nectar in plantations, forests and gardens in the wet, cold and dry season. Figure S4. Pollen and sucrose intake in plantations, forests and gardens in the wet, cold and dry season. Data S2. Results of generalized linear mixed effect models (exported from R Statistics). [file ECE3-6-1304-s001.doc]

**Supplemmentary Material**

Table S1

**Location of study sites and geographic information**. Due to limited space in gardens, garden sites were split into two locations. Landscape cover is given for the main landscape types studied (i.e. forest, plantation, garden). Latitude and longitude were rounded to the nearest minute to safeguard data privacy of land owners. Original data is available upon request.

| **Landscape** | **Study site** | **Region** | **Altitude**  **(a.s.l.)** | **Forest**  **cover [%]** | **Plantation**  **cover [%]** | **Garden**  **cover [%]** | **Latitude** | **Longitude** |
| --- | --- | --- | --- | --- | --- | --- | --- | --- |
| Forest | F1 | Bundaberg | 23 m | 96.36 |  |  | 24°38' S | 152° 8' E |
| Forest | F2 | Bundaberg | 27 m | 89.79 |  |  | 24°46' S | 152° 6' E |
| Forest | F3 | Sunshine Coast / Brisbane | 51 m | 97.52 |  |  | 26°43' S | 153° 1' E |
| Forest | F4 | Sunshine Coast / Brisbane | 83 m | 75.06 |  |  | 26°50' S | 152°55' E |
| Plantation | P1 | Bundaberg | 25 m |  | 94.73 |  | 24°46' S | 152°15' E |
| Plantation | P2 | Bundaberg | 27 m |  | 92.04 |  | 24°47' S | 152°15' E |
| Plantation | P3 | Bundaberg | 26 m |  | 93.21 |  | 24°47' S | 152°16' E |
| Plantation | P4 | Sunshine Coast / Brisbane | 55 m |  | 80.08 |  | 26°53' S | 152°56' E |
| Garden | G1 - location A | Bundaberg | 7 m |  |  | 88.20 | 24°50' S | 152°28' E |
| Garden | G1 - location B | Bundaberg | 19 m |  |  | 87.70 | 24°52' S | 152°19' E |
| Garden | G2 - location A | Sunshine Coast / Brisbane | 15 m |  |  | 98.46 | 26°41' S | 153° 7' E |
| Garden | G2 - location B | Sunshine Coast / Brisbane | 3 m |  |  | 76.99 | 26°41' S | 153° 7' E |
| Garden | G3 - location A | Sunshine Coast / Brisbane | 18 m |  |  | 98.21 | 27°29' S | 153° 0' E |
| Garden | G3 - location B | Sunshine Coast / Brisbane | 17 m |  |  | 82.23 | 27°29' S | 153° 0' E |
| Garden | G4 - location A | Sunshine Coast / Brisbane | 31 m |  |  | 82.06 | 27°30' S | 153° 1' E |
| Garden | G4 - location B | Sunshine Coast / Brisbane | 45 m |  |  | 78.23 | 27°29' S | 153° 1' E |

Data S1

Influence of daytime.

To test whether foraging patterns changed during the day, foraging observations were performed in the morning (9:30 – 12:50) and in the afternoon (13:55 – 16:40) from September to October 2011. We found that the proportion of pollen and nectar foragers significantly decreased from morning to afternoon (GLMM: pollen: χ^2^= 26.60, *P* < 0.001, nectar: χ^2^ = 5.01, *P* = 0.03), whereas the proportion of foragers returning without any load increased over the course of the day (χ^2^= 31.56, *P* < 0.001). Decreasing pollen foraging from morning to afternoon is consistent with findings in other stingless bees . No differences were observed for the proportion of returning resin foragers over the day (χ^2^= 2.82, *P* = 0.09). We therefore chose to perform our foraging observations at the earliest possible time for each observation day, when bee colonies reached sufficient activity levels. As a temperature sensitive species, workers of *T. carbonaria* need an ambient temperature of at least 18°C to start foraging . Observations in the cold season therefore started as late as 11:30 when temperature peaked at midday. Consequently, overall observation times ranged from 7:30 – 15:30 across seasons.

References

Heard, T.A. & Hendrikz, J.K. (1993) Factors influencing flight activity of colonies of the stingless bee *Trigona carbonaria* (Hymenoptera, Apidae). *Australian Journal of Zoology,* **41,** 343-353.

Inoue, T., Salmah, S., Abbas, I. & Yusuf, E. (1985) Foraging behavior of individual workers and foraging dynamics of colonies of three Sumatran stingless bees. *Researches on Population Ecology,* **27,** 373-392.

Wallace, H.M. & Lee, D.J. (2010) Resin-foraging by colonies of *Trigona sapiens* and *T. hockingsi* (Hymenoptera: Apidae, Meliponini) and consequent seed dispersal of *Corymbia torelliana* (Myrtaceae). *Apidologie,* **41,** 428-435.

Table S2

**Spearman correlation matrix** with correlation coefficients (r_S_) for forager numbers and weather variables; significance levels as follows: * *P* < 0.05, ** *P* < 0.01, *** *P* < 0.001. Abbreviations: **Act**: foraging activity; **P_P_**: proportion of pollen foragers; **P_N_**: proportion of nectar foragers; **P_R_**: proportion of resin foragers; **P_n_**: proportion of foragers with no load; **Temp**: temperature; **Hum**: humidity; **Cloud**: cloud cover; **W_max_**: maximum wind speed; **W_avg_**: average wind speed; **W_gusts_**: number of wind gusts; **F_N_**: nectar foragers per minute; **F_P_**: pollen foragers per minute; **F_R_**: resin foragers per minute; **F_n_**: foragers with no load per minute.

|  | **Act** | **P_P_** | **P_N_** | **P_R_** | **P_n_** | **Temp** | **Hum** | **Cloud** | **W_max_** | **W_avg_** | **W_gusts_** | **F_N_** | **F_P_** | **F_R_** |
| --- | --- | --- | --- | --- | --- | --- | --- | --- | --- | --- | --- | --- | --- | --- |
| **P_P_** | 0.12 * |  |  |  |  |  |  |  |  |  |  |  |  |  |
| **P_N_** | 0.15 ** | -0.42 *** |  |  |  |  |  |  |  |  |  |  |  |  |
| **P_R_** | -0.13 * | -0.05 | -0.41 *** |  |  |  |  |  |  |  |  |  |  |  |
| **P_n_** | -0.19 *** | -0.41 *** | -0.42 *** | 0.05 |  |  |  |  |  |  |  |  |  |  |
| **Temp** | 0.05 | 0 | 0.35 *** | -0.05 | -0.39 *** |  |  |  |  |  |  |  |  |  |
| **Hum** | 0.16 ** | -0.10 | 0.02 | 0.04 | 0.06 | -0.22 *** |  |  |  |  |  |  |  |  |
| **Cloud** | 0.11 * | -0.05 | 0.07 | 0.07 | -0.08 | 0.13 * | 0.42 *** |  |  |  |  |  |  |  |
| **W_max_** | -0.27 *** | -0.08 | -0.04 | -0.12 | 0.09 | 0.20 ** | -0.16 * | 0.08 |  |  |  |  |  |  |
| **W_avg_** | -0.25 *** | -0.12 | -0.01 | -0.09 | 0.11 | 0.19 * | -0.06 | 0.09 | 0.92 *** |  |  |  |  |  |
| **W_gusts_** | -0.22 ** | -0.01 | -0.12 | -0.10 | 0.10 | 0.09 | -0.02 | 0.11 | 0.82 *** | 0.90 *** |  |  |  |  |
| **F_N_** | 0.75 *** | -0.15 ** | 0.70 *** | -0.35 *** | -0.36 *** | 0.23 *** | 0.13 * | 0.15 ** | -0.22 ** | -0.20 ** | -0.23 ** |  |  |  |
| **F_P_** | 0.52 *** | 0.85 *** | -0.29 *** | 0 | -0.38 *** | 0.06 | -0.04 | -0.03 | -0.17 * | -0.20 ** | -0.12 | 0.19 *** |  |  |
| **F_R_** | 0.45 *** | 0.06 | -0.28 *** | 0.72 *** | -0.06 | -0.01 | 0.14 * | 0.09 | -0.30 *** | -0.30 *** | -0.30 *** | 0.12 ** | 0.30 *** |  |
| **F_n_** | 0.72 *** | -0.14 ** | -0.14 ** | -0.07 | 0.46 *** | -0.17 ** | 0.18 ** | 0.06 | -0.22 ** | -0.20 ** | -0.18 * | 0.41 *** | 0.21 *** | 0.37 *** |

**Table S3**

**Results of generalized linear mixed effect models** (GLMMs) for each response variable, for the second year with all weather factors included as additional explanatory variables. Given are χ^2^-values obtained for comparing the best model with the respective explanatory variable to a model with this variable dropped (landscape, season, temperature or wind). Significance levels as follows: * *P* < 0.05, ** *P* < 0.01, *** *P* < 0.001, ns: not significant. NA indicates missing data.

| Response variable | Landscape | | |  | Season | | |  | Temperature | | |  | Wind | | |
| --- | --- | --- | --- | --- | --- | --- | --- | --- | --- | --- | --- | --- | --- | --- | --- |
|  | *χ^2^* | *df* | *P* |  | *χ^2^* | *df* | *P* |  | *χ^2^* | *df* | *P* |  | *χ^2^* | *df* | *P* |
| foraging activity | 55.04 | 24 | *** |  | 54.18 | 24 | *** |  | 41.48 | 18 | *** |  | 35.43 | 18 | ** |
| pollen foragers / min | 375.87 | 24 | *** |  | 460.25 | 24 | *** |  | 276.53 | 18 | *** |  | 159.37 | 18 | *** |
| nectar foragers / min | 255.96 | 24 | *** |  | 293.72 | 24 | *** |  | 119.67 | 18 | *** |  | 241.74 | 18 | *** |
| resin foragers / min | 47.19 | 12 | *** |  | 66.34 | 12 | *** |  | 49.74 | 9 | ** |  |  |  | ns |
| unsuccessful foragers /min | 165.00 | 24 | *** |  | 168.76 | 24 | *** |  | 87.53 | 18 | *** |  | 105.45 | 18 | *** |
| proportion pollen foragers | 133.02 | 24 | *** |  | 153.90 | 24 | *** |  | 91.81 | 18 | *** |  | 43.51 | 18 | *** |
| proportion nectar foragers | 88.67 | 24 | *** |  | 138.16 | 24 | *** |  | 97.14 | 18 | *** |  | 62.72 | 18 | *** |
| proportion resin foragers | 42.90 | 12 | *** |  | 70.95 | 12 | *** |  |  |  | ns |  | 40.48 | 9 | *** |
| proportion unsuccessful foragers | 125.64 | 24 | *** |  | 138.28 | 24 | *** |  | 95.84 | 18 | *** |  | 74.71 | 18 | *** |
| total sugar intake / min | 189374 | 12 | *** |  | 259112 | 12 | *** |  | 251930 | 9 | *** |  | NA |  |  |
| total pollen intake / min | 59.47 | 12 | *** |  | 71.42 | 12 | *** |  | 59.03 | 9 | *** |  | NA |  |  |

Figure S1

**
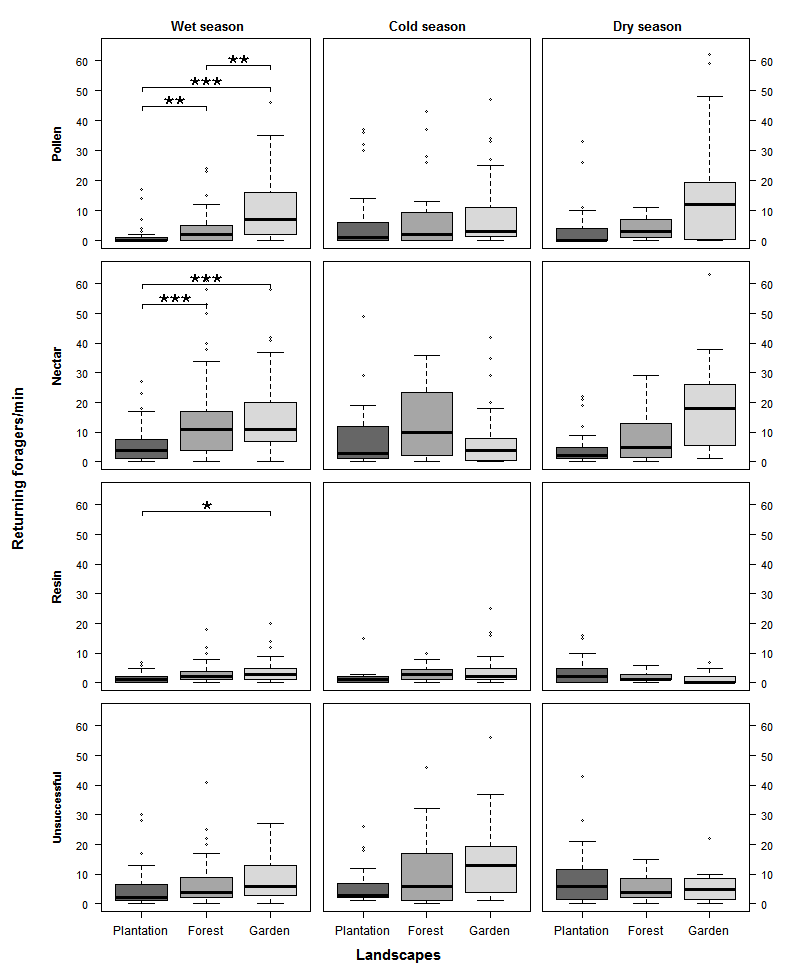
**

**Number of foragers returning per minute** with pollen, nectar, resin or unsuccessful foragers in plantations (dark gray bars), forests (gray) and gardens (light gray) in the wet, cold and dry season. Boxplots display the median (thick bar), lower (0.25) and upper (0.75) quartile (gray box), minimum and maximum values (whiskers) and outliers of each dataset. Asterisks indicate significant differences between landscapes according to Tukey’s posthoc test, significance levels as follows: * *P* < 0.05, ** *P* < 0.01, *** *P* < 0.001.

Figure S2


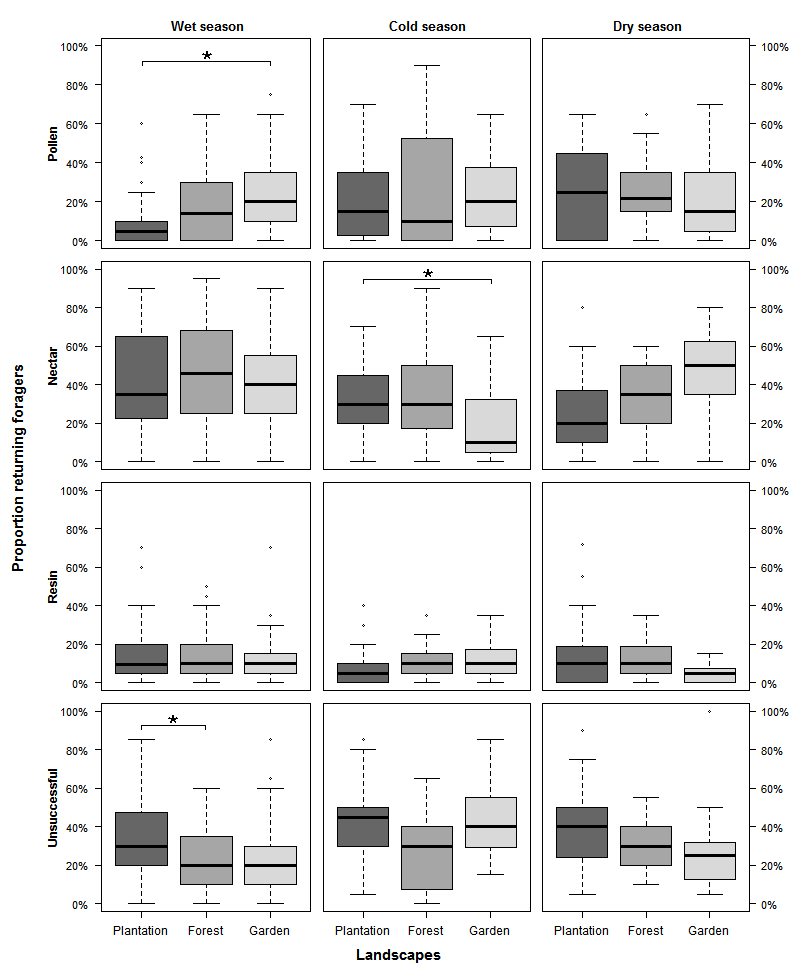


**Proportional resource intake** in plantations (dark gray bars), forests (gray) and gardens (light gray) in the wet, cold and dry season. Shown are percentages of foragers returning with pollen, nectar, resin and unsuccessful foragers in the wet, cold and dry season. Boxplots display the median (thick bar), lower (0.25) and upper (0.75) quartile (gray box), minimum and maximum values (whiskers) and outliers of each dataset. Asterisks indicate significant differences between landscapes according to Tukey’s posthoc test, significance levels as follows: * *P* < 0.05, ** *P* < 0.01, *** *P* < 0.001.

Figure S3


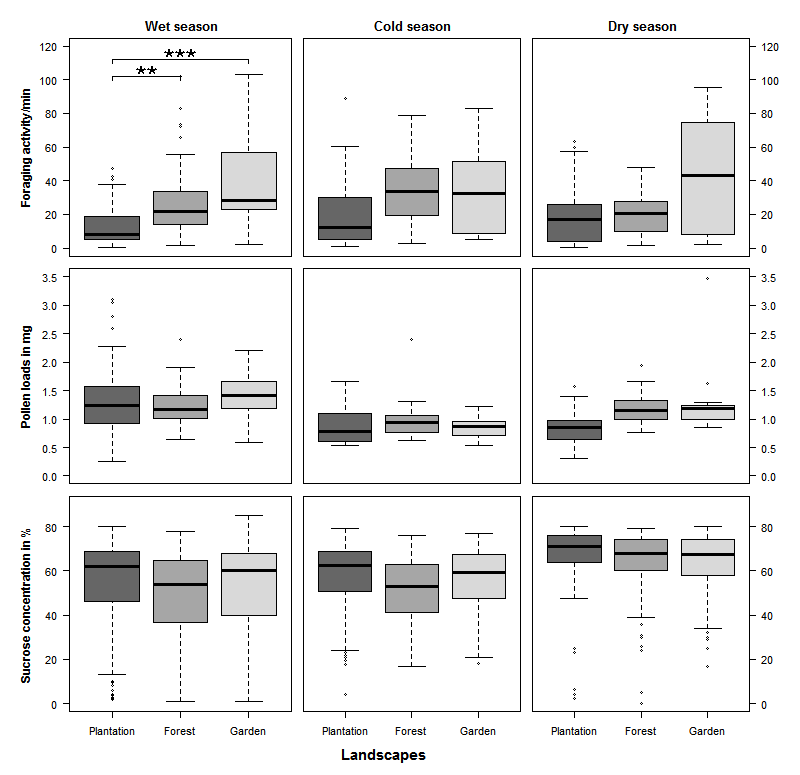


**Hive foraging activity, pollen loads and sucrose concentration** of nectar in plantations (dark gray bars), forests (gray) and gardens (light gray) in the wet, cold and dry season. Boxplots display the median (thick bar), lower (0.25) and upper (0.75) quartile (gray box), minimum and maximum values (whiskers) and outliers of each dataset. Asterisks indicate significant differences between landscapes according to Tukey’s posthoc test, significance levels as follows: * *P* < 0.05, ** *P* < 0.01, *** *P* < 0.001.

Figure S4


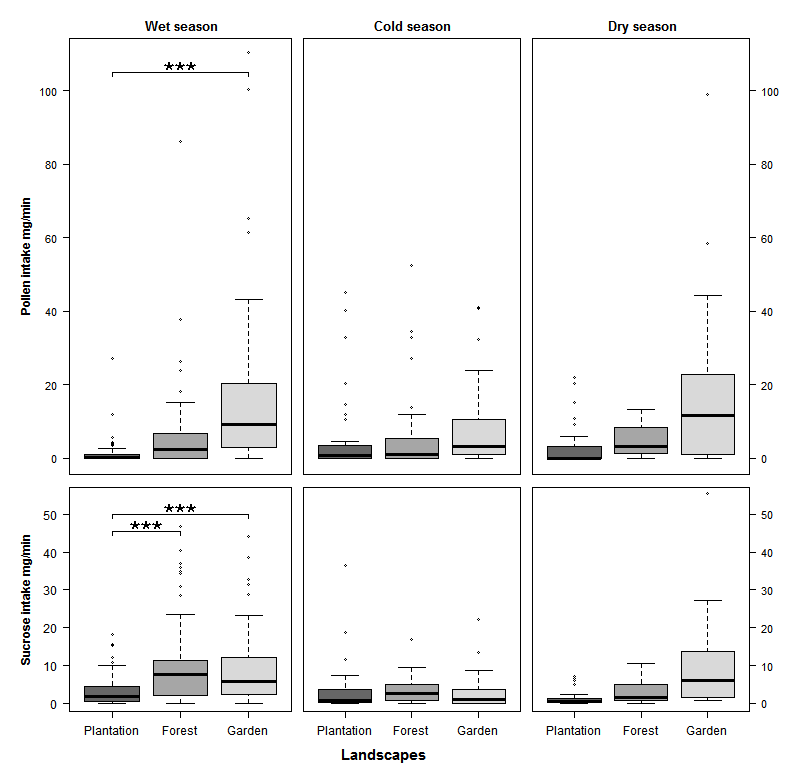


Pollen and sucrose intake in plantations (dark gray bars), forests (gray) and gardens (light gray) in the wet, cold and dry season. Boxplots display the median (thick bar), lower (0.25) and upper (0.75) quartile (gray box), minimum and maximum values (whiskers) and outliers of each dataset. Asterisks indicate significant differences between landscapes according to Tukey’s posthoc test, significance levels as follows: * P < 0.05, ** P < 0.01, *** P < 0.001.

**Data S2**

**Results of generalized linear mixed effect models** (exported from R Statistics). Models show the effects of landscape and season on various foraging parameters in the stingless bee *Tetragonula carbonaria*.

**Activity**

> summary(model_activity)

Linear mixed model fit by REML

Formula: activity_l_sqrt ~ landscape + (1 | site/as.factor(activity_data$hive))

Data: activity_data

AIC BIC logLik deviance REMLdev

1459 1482 -723.3 1449 1447

Random effects:

Groups Name Variance Std.Dev.

as.factor(activity_data$hive):site (Intercept) 0.0000 0.0000

site (Intercept) 1.1192 1.0579

Residual 2.8604 1.6913

Number of obs: 366, groups: as.factor(activity_data$hive):site, 48; site, 12

Fixed effects:

Estimate Std. Error t value

(Intercept) 3.2605 0.5635 5.787

landscape_F 1.5693 0.7950 1.974

landscape_G 2.5217 0.7992 3.155

Correlation of Fixed Effects:

(Intr) lands_F

landscape_F -0.709

landscape_G -0.705 0.500

**Pollen foragers / min**

> summary(model_forager)

Generalized linear mixed model fit by the Laplace approximation

Formula: For_a ~ landscape * season_total + (1 | site/as.factor(activity_data$hive))

Data: activity_data

AIC BIC logLik deviance

2549 2591 -1263 2527

Random effects:

Groups Name Variance Std.Dev.

as.factor(activity_data$hive):site (Intercept) 0.66363 0.81464

site (Intercept) 0.22739 0.47685

Number of obs: 366, groups: as.factor(activity_data$hive):site, 48; site, 12

Fixed effects:

Estimate Std. Error z value Pr(>|z|)

(Intercept) 0.6396 0.3599 1.777 0.07554 .

landscape_F 0.3462 0.4932 0.702 0.48274

landscape_G 2.1401 0.4837 4.424 9.67e-06 ***

season_wet -1.1453 0.1473 -7.777 7.43e-15 ***

season_cold 0.3146 0.1166 2.697 0.00699 **

landscape_F:season_wet 1.0787 0.1948 5.537 3.08e-08 ***

landscape_G:season_wet 0.4705 0.1722 2.732 0.00630 **

landscape_F:season_cold 0.2694 0.1735 1.553 0.12042

landscape_G:season_cold -1.0811 0.1502 -7.197 6.15e-13 ***

---

Signif. codes: 0 ‘***’ 0.001 ‘**’ 0.01 ‘*’ 0.05 ‘.’ 0.1 ‘ ’ 1

Correlation of Fixed Effects:

(Intr) lands_F lands_G seas_wet seas_col lands_F:seas_wet lands_G:seas_wet lands_F:seas_col

landscape_F -0.730

landscape_G -0.744 0.543

season_wet -0.194 0.141 0.144

season_cold -0.195 0.143 0.145 0.500

lands_F:seas_wet 0.146 -0.246 -0.109 -0.756 -0.378

lands_G:seas_wet 0.165 -0.121 -0.200 -0.855 -0.427 0.646

lands_F:seas_col 0.131 -0.224 -0.098 -0.336 -0.672 0.575 0.287

lands_G:seas_col 0.152 -0.111 -0.185 -0.388 -0.776 0.293 0.530 0.522

**Nectar foragers / min**

> summary(model_forager)

Generalized linear mixed model fit by the Laplace approximation

Formula: For_a ~ landscape * season_total + (1 | site/as.factor(activity_data$hive))

Data: activity_data

AIC BIC logLik deviance

2963 3006 -1470 2941

Random effects:

Groups Name Variance Std.Dev.

as.factor(activity_data$hive):site (Intercept) 0.14985 0.38711

site (Intercept) 0.11623 0.34092

Number of obs: 366, groups: as.factor(activity_data$hive):site, 48; site, 12

Fixed effects:

Estimate Std. Error z value Pr(>|z|)

(Intercept) 1.1531 0.2248 5.129 2.92e-07 ***

landscape_F 0.7314 0.3086 2.370 0.017786 *

landscape_G 1.9093 0.3075 6.209 5.33e-10 ***

season_wet 0.3460 0.1014 3.411 0.000646 ***

season_cold 0.5323 0.1078 4.939 7.86e-07 ***

landscape_F:season_wet 0.2335 0.1306 1.788 0.073742 .

landscape_G:season_wet -0.7672 0.1302 -5.893 3.79e-09 ***

landscape_F:season_cold -0.1799 0.1401 -1.284 0.199054

landscape_G:season_cold -1.3774 0.1431 -9.624 < 2e-16 ***

---

Signif. codes: 0 ‘***’ 0.001 ‘**’ 0.01 ‘*’ 0.05 ‘.’ 0.1 ‘ ’ 1

Correlation of Fixed Effects:

(Intr) lands_F lands_G seas_wet seas_col lands_F:seas_wet lands_G:seas_wet lands_F:seas_col

landscape_F -0.729

landscape_G -0.731 0.533

season_wet -0.353 0.257 0.258

season_cold -0.308 0.224 0.225 0.693

lands_F:seas_wet 0.274 -0.349 -0.200 -0.777 -0.538

lands_G:seas_wet 0.275 -0.200 -0.339 -0.779 -0.540 0.605

lands_F:seas_col 0.237 -0.297 -0.173 -0.533 -0.769 0.707 0.415

lands_G:seas_col 0.232 -0.169 -0.277 -0.522 -0.753 0.405 0.651 0.579

**Resin foragers / min**

> summary(model_forager)

Generalized linear mixed model fit by the Laplace approximation

Formula: For_a ~ landscape * season_total + (1 | site/as.factor(activity_data$hive))

Data: activity_data

AIC BIC logLik deviance

970.6 1014 -474.3 948.6

Random effects:

Groups Name Variance Std.Dev.

as.factor(activity_data$hive):site (Intercept) 0.21301 0.46153

site (Intercept) 0.33580 0.57948

Number of obs: 366, groups: as.factor(activity_data$hive):site, 48; site, 12

Fixed effects:

Estimate Std. Error z value Pr(>|z|)

(Intercept) 0.6522 0.3524 1.850 0.0642 .

landscape_F -0.1723 0.4988 -0.346 0.7297

landscape_G -0.8150 0.5292 -1.540 0.1235

season_wet -0.7831 0.1420 -5.516 3.47e-08 ***

season_cold -0.9863 0.1750 -5.637 1.73e-08 ***

landscape_F:season_wet 1.2056 0.2206 5.466 4.61e-08 ***

landscape_G:season_wet 2.0332 0.2792 7.282 3.30e-13 ***

landscape_F:season_cold 1.3649 0.2509 5.441 5.30e-08 ***

landscape_G:season_cold 2.0386 0.3026 6.737 1.61e-11 ***

---

Signif. codes: 0 ‘***’ 0.001 ‘**’ 0.01 ‘*’ 0.05 ‘.’ 0.1 ‘ ’ 1

Correlation of Fixed Effects:

(Intr) lands_F lands_G seas_wet seas_col lands_F:seas_wet lands_G:seas_wet lands_F:seas_col

landscape_F -0.707

landscape_G -0.666 0.471

season_wet -0.227 0.161 0.151

season_cold -0.147 0.104 0.098 0.380

lands_F:seas_wet 0.146 -0.330 -0.097 -0.644 -0.245

lands_G:seas_wet 0.116 -0.082 -0.447 -0.508 -0.193 0.327

lands_F:seas_col 0.103 -0.246 -0.068 -0.265 -0.697 0.562 0.135

lands_G:seas_col 0.085 -0.060 -0.386 -0.220 -0.578 0.142 0.733 0.403

**Unsuccessful foragers / min**

> summary(model_forager)

Generalized linear mixed model fit by the Laplace approximation

Formula: For_a ~ landscape * season_total + (1 | site/as.factor(activity_data$hive))

Data: activity_data

AIC BIC logLik deviance

1534 1577 -755.9 1512

Random effects:

Groups Name Variance Std.Dev.

as.factor(activity_data$hive):site (Intercept) 0.068885 0.26246

site (Intercept) 0.369838 0.60814

Number of obs: 366, groups: as.factor(activity_data$hive):site, 48; site, 12

Fixed effects:

Estimate Std. Error z value Pr(>|z|)

(Intercept) 1.66014 0.32767 5.066 4.05e-07 ***

landscape_F -0.34368 0.46428 -0.740 0.459

landscape_G -0.23717 0.46760 -0.507 0.612

season_wet -0.54834 0.08451 -6.488 8.69e-11 ***

season_cold -0.47989 0.09365 -5.125 2.98e-07 ***

landscape_F:season_wet 0.72380 0.13459 5.378 7.53e-08 ***

landscape_G:season_wet 0.99684 0.14853 6.712 1.93e-11 ***

landscape_F:season_cold 1.07364 0.14077 7.627 2.41e-14 ***

landscape_G:season_cold 1.49531 0.15131 9.883 < 2e-16 ***

---

Signif. codes: 0 ‘***’ 0.001 ‘**’ 0.01 ‘*’ 0.05 ‘.’ 0.1 ‘ ’ 1

Correlation of Fixed Effects:

(Intr) lands_F lands_G seas_wet seas_col lands_F:seas_wet lands_G:seas_wet lands_F:seas_col

landscape_F -0.706

landscape_G -0.701 0.495

season_wet -0.164 0.116 0.115

season_cold -0.119 0.084 0.083 0.478

lands_F:seas_wet 0.103 -0.222 -0.072 -0.628 -0.300

lands_G:seas_wet 0.093 -0.066 -0.256 -0.569 -0.272 0.357

lands_F:seas_col 0.079 -0.175 -0.055 -0.318 -0.665 0.609 0.181

lands_G:seas_col 0.073 -0.052 -0.218 -0.296 -0.619 0.186 0.688 0.412

**Proportion of pollen foragers**

> summary(model_prop)

Generalized linear mixed model fit by the Laplace approximation

Formula: prop ~ landscape * season_total + (1 | site/as.factor(proportion$hive))

Data: proportion

AIC BIC logLik deviance

1696 1740 -836.8 1674

Random effects:

Groups Name Variance Std.Dev.

as.factor(proportion$hive):site (Intercept) 0.481790 0.69411

site (Intercept) 0.093858 0.30636

Number of obs: 414, groups: as.factor(proportion$hive):site, 49; site, 12

Fixed effects:

Estimate Std. Error z value Pr(>|z|)

(Intercept) -1.40694 0.26392 -5.331 9.77e-08 ***

landscape_F -0.03722 0.36537 -0.102 0.918862

landscape_G 0.32456 0.37454 0.867 0.386179

season_wet -1.06527 0.12499 -8.523 < 2e-16 ***

season_cold -0.19096 0.12293 -1.553 0.120329

landscape_F:season_wet 0.75939 0.17611 4.312 1.62e-05 ***

landscape_G:season_wet 0.93296 0.19393 4.811 1.50e-06 ***

landscape_F:season_cold 0.59049 0.17869 3.305 0.000951 ***

landscape_G:season_cold -0.02848 0.19942 -0.143 0.886436

---

Signif. codes: 0 ‘***’ 0.001 ‘**’ 0.01 ‘*’ 0.05 ‘.’ 0.1 ‘ ’ 1

Correlation of Fixed Effects:

(Intr) lands_F lands_G seas_wet seas_col lands_F:seas_wet lands_G:seas_wet lands_F:seas_col

landscape_F -0.722

landscape_G -0.705 0.509

season_wet -0.228 0.165 0.161

season_cold -0.182 0.132 0.128 0.393

lands_F:seas_wet 0.162 -0.287 -0.114 -0.710 -0.279

lands_G:seas_wet 0.147 -0.106 -0.341 -0.645 -0.253 0.457

lands_F:seas_col 0.125 -0.204 -0.088 -0.271 -0.688 0.429 0.174

lands_G:seas_col 0.112 -0.081 -0.274 -0.242 -0.616 0.172 0.527 0.424

**Proportion of nectar foragers**

> summary(model_prop)

Generalized linear mixed model fit by the Laplace approximation

Formula: prop ~ landscape * season_total + (1 | site/as.factor(proportion$hive))

Data: proportion

AIC BIC logLik deviance

1768 1812 -872.9 1746

Random effects:

Groups Name Variance Std.Dev.

as.factor(proportion$hive):site (Intercept) 0.154868 0.39353

site (Intercept) 0.060565 0.24610

Number of obs: 414, groups: as.factor(proportion$hive):site, 49; site, 12

Fixed effects:

Estimate Std. Error z value Pr(>|z|)

(Intercept) -1.18047 0.18419 -6.409 1.47e-10 ***

landscape_F 0.52127 0.25790 2.021 0.04326 *

landscape_G 1.36665 0.26666 5.125 2.97e-07 ***

season_wet 0.82250 0.09733 8.450 < 2e-16 ***

season_cold 0.35407 0.11360 3.117 0.00183 **

landscape_F:season_wet -0.32605 0.14019 -2.326 0.02003 *

landscape_G:season_wet -1.36592 0.15745 -8.675 < 2e-16 ***

landscape_F:season_cold -0.50418 0.16141 -3.124 0.00179 **

landscape_G:season_cold -1.57039 0.18151 -8.652 < 2e-16 ***

---

Signif. codes: 0 ‘***’ 0.001 ‘**’ 0.01 ‘*’ 0.05 ‘.’ 0.1 ‘ ’ 1

Correlation of Fixed Effects:

(Intr) lands_F lands_G seas_wet seas_col lands_F:seas_wet lands_G:seas_wet lands_F:seas_col

landscape_F -0.714

landscape_G -0.691 0.493

season_wet -0.367 0.262 0.253

season_cold -0.277 0.198 0.192 0.532

lands_F:seas_wet 0.255 -0.395 -0.176 -0.694 -0.369

lands_G:seas_wet 0.227 -0.162 -0.440 -0.618 -0.329 0.429

lands_F:seas_col 0.195 -0.277 -0.135 -0.375 -0.704 0.521 0.232

lands_G:seas_col 0.174 -0.124 -0.323 -0.333 -0.626 0.231 0.536 0.440

**Proportion of resin foragers**

> summary(model_prop)

Generalized linear mixed model fit by the Laplace approximation

Formula: prop ~ landscape * season_total + (1 | site/as.factor(proportion$hive))

Data: proportion

AIC BIC logLik deviance

1046 1090 -511.9 1024

Random effects:

Groups Name Variance Std.Dev.

as.factor(proportion$hive):site (Intercept) 0.19004 0.43594

site (Intercept) 0.15235 0.39032

Number of obs: 414, groups: as.factor(proportion$hive):site, 49; site, 12

Fixed effects:

Estimate Std. Error z value Pr(>|z|)

(Intercept) -1.7761 0.2537 -7.001 2.55e-12 ***

landscape_F -0.3064 0.3639 -0.842 0.3999

landscape_G -1.6359 0.4131 -3.960 7.48e-05 ***

season_wet -0.0735 0.1282 -0.573 0.5665

season_cold -0.7177 0.1747 -4.108 3.99e-05 ***

landscape_F:season_wet 0.2777 0.1984 1.400 0.1615

landscape_G:season_wet 1.2815 0.2730 4.694 2.68e-06 ***

landscape_F:season_cold 0.6398 0.2453 2.608 0.0091 **

landscape_G:season_cold 1.6301 0.3081 5.291 1.22e-07 ***

---

Signif. codes: 0 ‘***’ 0.001 ‘**’ 0.01 ‘*’ 0.05 ‘.’ 0.1 ‘ ’ 1

Correlation of Fixed Effects:

(Intr) lands_F lands_G seas_wet seas_col lands_F:seas_wet lands_G:seas_wet lands_F:seas_col

landscape_F -0.697

landscape_G -0.614 0.428

season_wet -0.323 0.225 0.198

season_cold -0.200 0.139 0.123 0.406

lands_F:seas_wet 0.209 -0.389 -0.128 -0.646 -0.262

lands_G:seas_wet 0.152 -0.106 -0.550 -0.470 -0.191 0.304

lands_F:seas_col 0.142 -0.245 -0.087 -0.289 -0.712 0.464 0.136

lands_G:seas_col 0.113 -0.079 -0.446 -0.230 -0.567 0.149 0.670 0.404

**Proportion of unsuccessful foragers**

> summary(model_prop)

Generalized linear mixed model fit by the Laplace approximation

Formula: prop ~ landscape * season_total + (1 | site/as.factor(proportion$hive))

Data: proportion

AIC BIC logLik deviance

1315 1359 -646.3 1293

Random effects:

Groups Name Variance Std.Dev.

as.factor(proportion$hive):site (Intercept) 0.073002 0.27019

site (Intercept) 0.039313 0.19828

Number of obs: 414, groups: as.factor(proportion$hive):site, 49; site, 12

Fixed effects:

Estimate Std. Error z value Pr(>|z|)

(Intercept) -0.52873 0.14537 -3.637 0.000276 ***

landscape_F -0.29187 0.21154 -1.380 0.167664

landscape_G -0.90446 0.22397 -4.038 5.38e-05 ***

season_wet -0.10878 0.09066 -1.200 0.230218

season_cold 0.12967 0.10310 1.258 0.208488

landscape_F:season_wet -0.42379 0.14324 -2.959 0.003091 **

landscape_G:season_wet 0.26478 0.16016 1.653 0.098291 .

landscape_F:season_cold -0.26426 0.15700 -1.683 0.092344 .

landscape_G:season_cold 0.87028 0.16969 5.129 2.92e-07 ***

---

Signif. codes: 0 ‘***’ 0.001 ‘**’ 0.01 ‘*’ 0.05 ‘.’ 0.1 ‘ ’ 1

Correlation of Fixed Effects:

(Intr) lands_F lands_G seas_wet seas_col lands_F:seas_wet lands_G:seas_wet lands_F:seas_col

landscape_F -0.687

landscape_G -0.649 0.446

season_wet -0.391 0.268 0.254

season_cold -0.296 0.203 0.192 0.483

lands_F:seas_wet 0.247 -0.453 -0.160 -0.633 -0.306

lands_G:seas_wet 0.221 -0.152 -0.515 -0.566 -0.273 0.358

lands_F:seas_col 0.194 -0.316 -0.126 -0.317 -0.657 0.477 0.180

lands_G:seas_col 0.180 -0.124 -0.412 -0.293 -0.608 0.186 0.564 0.399

**Pollen load size**

> summary (model_loads)

Linear mixed model fit by REML

Formula: loads ~ season_total + (1 | site/as.factor(loads_data$hive))

Data: loads_data

AIC BIC logLik deviance REMLdev

349.2 371.6 -168.6 325.8 337.2

Random effects:

Groups Name Variance Std.Dev.

as.factor(loads_data$hive):site (Intercept) 0.0042788 0.065413

site (Intercept) 0.0350893 0.187321

Residual 0.1564168 0.395496

Number of obs: 307, groups: as.factor(loads_data$hive):site, 51; site, 12

Fixed effects:

Estimate Std. Error t value

(Intercept) 1.13301 0.08045 14.083

season_wet 0.19143 0.06342 3.018

season_cold -0.19940 0.07067 -2.821

Correlation of Fixed Effects:

(Intr) seas_wet

season_wet -0.638

season_cold -0.495 0.628

**Pollen intake (mg / min)**

> summary(model_pollen)

Generalized linear mixed model fit by the Laplace approximation

Formula: pollen ~ landscape + season_total + (1 | site/as.factor(pollen_total_data$hive))

Data: pollen_total_data

AIC BIC logLik deviance

1364 1391 -674.8 1350

Random effects:

Groups Name Variance Std.Dev.

as.factor(pollen_total_data$hive):site (Intercept) 0.163616 0.40449

site (Intercept) 0.034804 0.18656

Number of obs: 366, groups: as.factor(pollen_total_data$hive):site, 48; site, 12

Fixed effects:

Estimate Std. Error z value Pr(>|z|)

(Intercept) 1.34266 0.16133 8.322 < 2e-16 ***

landscape_F 0.45453 0.21187 2.145 0.031925 *

landscape_G 0.78638 0.21104 3.726 0.000194 ***

season_wet -0.14157 0.06161 -2.298 0.021568 *

season_cold -0.21523 0.06791 -3.169 0.001528 **

---

Signif. codes: 0 ‘***’ 0.001 ‘**’ 0.01 ‘*’ 0.05 ‘.’ 0.1 ‘ ’ 1

Correlation of Fixed Effects:

(Intr) lands_F lands_G seas_wet

landscape_F -0.695

landscape_G -0.696 0.539

season_wet -0.268 -0.032 -0.037

season_cold -0.220 -0.010 -0.028 0.623

**Nectar concentration**

> summary(model_concentration)

Linear mixed model fit by REML

Formula: concentration ~ landscape * season_total + (1 | site/as.factor(conc_data$hive))

Data: conc_data

AIC BIC logLik deviance REMLdev

22336 22406 -11156 22339 22312

Random effects:

Groups Name Variance Std.Dev.

as.factor(conc_data$hive):site (Intercept) 12.204 3.4934

site (Intercept) 30.185 5.4941

Residual 263.589 16.2354

Number of obs: 2647, groups: as.factor(conc_data$hive):site, 53; site, 12

Fixed effects:

Estimate Std. Error t value

(Intercept) 68.916 3.043 22.651

landscape_F -0.503 3.885 -0.130

landscape_G -7.592 4.305 -1.763

season_wet -10.944 1.580 -6.926

season_cold -6.189 1.828 -3.385

landscape_F:season_wet -7.336 2.227 -3.294

landscape_G:season_wet 1.981 2.449 0.809

landscape_F:season_cold -5.224 2.557 -2.043

landscape_G:season_cold -1.843 2.752 -0.670

Correlation of Fixed Effects:

(Intr) lands_F lands_G seas_wet seas_col lands_F:seas_wet lands_G:seas_wet lands_F:seas_col

landscape_F -0.676

landscape_G -0.691 0.529

season_wet -0.442 0.357 0.314

season_cold -0.346 0.272 0.245 0.673

lands_F:seas_wet 0.305 -0.494 -0.211 -0.710 -0.478

lands_G:seas_wet 0.281 -0.245 -0.450 -0.646 -0.434 0.457

lands_F:seas_col 0.250 -0.381 -0.177 -0.481 -0.715 0.661 0.311

lands_G:seas_col 0.229 -0.184 -0.337 -0.447 -0.664 0.317 0.599 0.475

**Sugar intake (mg / min)**

> summary(model_sugar)

Generalized linear mixed model fit by the Laplace approximation

Formula: sugar ~ landscape * season_total + (1 | site/as.factor(sugar_data$hive))

Data: sugar_data

AIC BIC logLik deviance

1899619 1899662 -949799 1899597

Random effects:

Groups Name Variance Std.Dev.

as.factor(sugar_data$hive):site (Intercept) 0.274801 0.52421

site (Intercept) 0.062475 0.24995

Number of obs: 366, groups: as.factor(sugar_data$hive):site, 48; site, 12

Fixed effects:

Estimate Std. Error z value Pr(>|z|)

(Intercept) 6.892722 0.189490 36.38 < 2e-16 ***

landscape_F 0.981429 0.260355 3.77 0.000164 ***

landscape_G 2.688392 0.260819 10.31 < 2e-16 ***

season_wet 1.025930 0.005464 187.75 < 2e-16 ***

season_cold 0.997624 0.005853 170.44 < 2e-16 ***

landscape_F:season_wet 0.146078 0.006782 21.54 < 2e-16 ***

landscape_G:season_wet -1.635497 0.006530 -250.46 < 2e-16 ***

landscape_F:season_cold -0.828214 0.007529 -110.00 < 2e-16 ***

landscape_G:season_cold -2.318527 0.007370 -314.60 < 2e-16 ***

---

Signif. codes: 0 ‘***’ 0.001 ‘**’ 0.01 ‘*’ 0.05 ‘.’ 0.1 ‘ ’ 1

Correlation of Fixed Effects:

(Intr) lands_F lands_G seas_wet seas_col lands_F:seas_wet lands_G:seas_wet lands_F:seas_col

landscape_F -0.728

landscape_G -0.727 0.529

season_wet -0.025 0.018 0.018

season_cold -0.023 0.016 0.016 0.786

lands_F:seas_wet 0.020 -0.023 -0.015 -0.806 -0.634

lands_G:seas_wet 0.021 -0.015 -0.021 -0.837 -0.658 0.674

lands_F:seas_col 0.018 -0.020 -0.013 -0.611 -0.777 0.768 0.512

lands_G:seas_col 0.018 -0.013 -0.018 -0.625 -0.794 0.503 0.703 0.617
